# Administration of Ether—Boston Rules

**Published:** 1874-01

**Authors:** Henry J. Bigelow

**Affiliations:** Surgeon to Massachusetts General Hospital


Administration of Ether—*Henry J. Bigelow, M. D., Surgeon to Massachusetts General Hospital.*—A few purely practical suggestions, in a familiar form, however superfluous or even trite to a part of the surgical world, may perhaps not inappropriately serve as a record of the current views and practice of etherization in the hospital with which I am connected—which has, perhaps, a larger experience than any other, of this form of anæsthesia.

1. Accept the odor and bulk of ether as a cheap compromise for the safety of the patient and the confidence it gives the operator.

2. Believe that its anæsthetic effects, whether pleasant or objectionable, do not differ materially from those of chloroform.

3. Recognize the fact, that, while chloroform may kill without warning, ether never does.

4. Aim at anæsthesia by inebriation, not by asphyxia. With ether vapor, insure air to the patient. Though he struggle at the beginning, if he is not rigid or too livid, it is safe to compel inhalation; but if you can devote more time to the process, the resistance will be often less.

5. Use, and let hospital assistants use, a good-sized bell-shaped sponge; and then it may be a question of less rather than more air. The various forms of apparatus which restrict or graduate the quantity of air require more attention and more assistance. Of these a close bag is the worst. If the sponge is damp, it retains ether better, while the vapor is perhaps a little softer than when absolutely pure. The ready ignition of the latter suggests the precaution of moistening with water the skin and saturated linen, before employing near the face even galvano-cautery.

6. Keep the pulse in hand; at any rate, examine it often. When the pulse is right, the patient is so. With chloroform, the pulse may be right and the patient wrong. If slow or feeble, or if the patient snores more than he need, save his strength by giving air—at any rate, until the pulse comes up; but renew the ether before he is sensible of pain. If the pulse shows that he is suddenly faint, lay him down and give him air.

7. If the patient is livid or rigid, give him air.

8. If his glottis contracts, give him air.

9. If he breathes badly, put the finger inside the cheek to admit air over the base of the tongue.

10. Should he vomit, of which there is usually timely notice, give the matters free exit by turning the patient, if recumbent, well to one side. Although there is less nausea with an empty stomach, it is not well to starve a patient about to encounter a protracted operation.

11. From time to time evacuate the tracheal mucus from the fauces, during an expiration, with a sponge held in dressing forceps.

12. In operations about the nose and mouth, give, for convenience, a powerful dose before beginning. Impregnate the whole circulation to the degree it usually attains in the middle of a long operation. The patient is then easily kept quiet. Otherwise a volume of fresh blood may find its way to the brain, and suddenly revive him. Let the repeated does be also heavy.

13. In these operations, expect blood in the trachea, and evacuate it like the mucus—but, by reason of its quantity, more promptly.

14. Indeed, if such an operation promises much blood, have a tracheotomy tube ready, with hooks to hold the incision open while they compress the veins, so that the tube can be entered by a cut or two in a few seconds.

15. Or insert the tube before the operation, and put a sponge in the pharynx. The patient may then be etherized through the tube. I have had occasion to resort to these expedients.

16. In artificial respiration, act with the patient, and not against him. He will not cease to breathe at once, and wholly. Enjoin silence; watch the first attempt at inspiration, and at the expiration compress the thorax, aiding its elastic reaction, if absolutely necessary, by Sylvester's, or other quiet method. See that the tongue is well forward.

17. Do not cool the patient by exposure and wet surroundings.

18. Being first assured that he can swallow a teaspoonful of water, feed him, if you like, with stimulus, during the expiration, but not the inspiration.

19. Give to all painful surgery, without exception, the benefit of anæsthesia; but a patient unequivocally exhausted by long disease—of the bladder, or of a joint, for example—or an habitual inebriate, may require care; without which, protracted narcotism may gradually depress his pulse beyond the rallying point. On the other hand, a healthy laborer, who reaches the hospital some hours after a railroad accident, cold, and literally pulseless at the wrist, from hemorrhage and exposure, is, as a rule, stimulated by ether, during and after at least one amputation.

20. Notwithstanding every expedient, there is occasionally an untoward subject who is habitually tetanic or livid, whenever etherized; or, more rarely, one whose respiration is notably intermittent before he becomes insensible. The latter requires attention. In children, it may be added, anæsthesia is cumulative.

Such are some of the minor considerations and prompt precautions which collectively determine the question of life or death in the exceptional emergencies of anæsthesia by ether. Many of them apply with equal force to chloroform; but against the shock of chloroform and its sequences, whether "chloroformic syncope," "cerebral anæmia," or "cerebral congestion," precaution avails nothing.

A Definition of Life.—The *Medical and Surgical Reporter* offers the following, which it states is "substantially, though not verbally, propounded by perhaps the profoundest living zoologist of this century, Cuvier, in his Animal Kingdom (vol. ii. p. 71): 'Life is that condition of being in which the form is more essential than the matter.'"

